# Costs and Cost Drivers of Providing Option B+ Services to Mother-Baby Pairs for PMTCT of HIV in Health Centre IV Facilities in Jinja District, Uganda

**DOI:** 10.1155/2020/2875864

**Published:** 2020-05-18

**Authors:** Aggrey D. Mukose, Senait Kebede, Christine Muhumuza, Fredrick Makumbi, Henry Komakech, Esther Bayiga, Denis Busobozi, Joshua Musinguzi, Andreas Kuznik, Peter Stegman, Steven Forsythe, Joseph Kagaayi

**Affiliations:** ^1^Department of Epidemiology and Biostatistics, School of Public Health, College of Health Sciences, Makerere University, Kampala, Uganda; ^2^UNAIDS, Uganda; ^3^Department of Community Health and Behavioral Sciences, School of Public Health, College of Health Sciences, Makerere University, Kampala, Uganda; ^4^International AIDS Economics Network (IAEN) and Avenir Health Costing Study, Makerere University School of Public Health, Kampala, Uganda; ^5^Uganda AIDS Commission, Uganda; ^6^Ministry of Health, STD/AIDS Control Program, Kampala, Uganda; ^7^Celgene Corporation, Summit, New Jersey, USA; ^8^Avenir Health, Washington DC, USA

## Abstract

**Background:**

In 2013, the World Health Organization (WHO) revised the 2012 guidelines on use of antiretroviral drugs (ARVs) for the prevention of mother-to-child transmission (PMTCT) of human immunodeficiency virus (HIV). The new guidelines recommended lifelong antiretroviral therapy (ART) for all HIV-positive pregnant and breastfeeding women irrespective of CD4 count or clinical stage (also referred to as Option B+). Uganda started implementing Option B+ in 2012 basing on the 2012 WHO guidelines. Despite the impressive benefits of the Option B+ strategy, implementation challenges, including cost burden and mother-baby pairs lost to follow-up, threatened its overall effectiveness. The researchers were unable to identify any studies conducted to assess costs and cost drivers associated with provision of Option B+ services to mother-baby pairs in HIV care in Uganda. Therefore, this study determined costs and cost drivers of providing Option B+ services to mother-baby pairs over a two-year period (2014–2015) in selected health facilities in Jinja district, Uganda.

**Methods:**

The estimated costs of providing Option B+ to mother-baby pairs derived from the provider perspective were evaluated at four health centres (HC) in Jinja district. A retrospective, ingredient-based costing approach was used to collect data for 2014 as base year using a standardized cost data capture tool. All costs were valued in United States dollars (USD) using the 2014 midyear exchange rate. Costs incurred in the second year (2015) were obtained by inflating the 2014 costs by the ratio of 2015 and 2014 USA Gross Domestic Product (GDP) implicit price deflator.

**Results:**

The average total cost of Option B+ services per HC was 66,512.7 (range: 32,168.2–102,831.1) USD over the 2-year period. The average unit cost of Option B+ services per mother-baby pair was USD 441.9 (range: 422.5–502.6). ART for mothers was the biggest driver of total mean costs (percent contribution: 62.6%; range: 56.0%–65.5%) followed by facility personnel (percent contribution: 8.2%; range: 7.7%–11.6%), and facility-level monitoring and quality improvement (percent contribution: 6.0%; range: 3.2%–12.3%). *Conclusions and Recommendations*. ART for mothers was the major cost driver. Efforts to lower the cost of ART for PMTCT would make delivery of Option B+ affordable and sustainable.

## 1. Background

Globally, HIV infection continues to pose a serious health risk for pregnant women and their children, especially in high HIV burden countries [1]. Mother-to-child transmission (MTCT) of HIV accounts for over 90% of new HIV infections among children. One in five new HIV infections is through preventable vertical transmission from the mother to the baby during pregnancy, labour, delivery, or breastfeeding [2]. In 2015, an estimated 150,000 children globally acquired HIV through MTCT. Of all these new infections, 88% occurred in sub-Saharan Africa [3]. In Uganda, MTCT of HIV accounted for 18% of new HIV infections [4]. In 2015, slightly over 95,000 children were estimated to be living with HIV in Uganda. Of these, 4% were new infections. In the same year, acquired immunodeficiency syndrome (AIDS) was responsible for 17% of deaths among children [5].

In 2013, the WHO revised the 2012 guidelines and made new recommendations on the use of ARVs for PMTCT of HIV [6, 7]. The revised guidelines included a new, third option (Option B+) which recommended lifelong ART for all HIV-positive pregnant and breastfeeding women irrespective of CD4 cell count or clinical stage. This recommendation was identified as high priority for countries with high HIV prevalence and fertility rates like Uganda [8, 9]. In 2012, Uganda had a total fertility rate of 6.2 (6.2 children per woman aged 15-49 years) and HIV prevalence of 7.3% [10]. Between 2009 and 2015, Uganda documented up to an 86% reduction in the number of new HIV infections in children indicating the great reduction of the MTCT as a public health threat [11]. Much of this progress is attributed to the adoption of Option B+ in 2012, which was initiated in the central districts of Uganda and was extended to the entire country by 2013. This led to a dramatic increase in the number of HIV-positive pregnant and breastfeeding women able to access ART. For example, the number of HIV-positive mothers who received ART for PMTCT increased from 112,909 in 2014 to 117,854 in June 2016 [5]. This increased access to ART led to both an improvement in the health of mothers and in a reduction of MTCT of HIV.

Sustaining the delivery of Option B+ requires an insight into and an appreciation of factors that support treatment, care, and retention of mothers and their babies in care which includes a strong understanding of costs and cost drivers of providing such services [12]. Health economic evaluations are important for understanding the cost implications for the service provider and clients, and can assist in determining effective approaches and informing healthcare resource allocation choices.

As advances in HIV treatment are embraced by national healthcare systems globally, it is essential for program managers and policy makers to know the long-term trade-offs between their costs and benefits. To this end, cost analysis of retention in Option B+ programs has been conducted for generalized epidemics in low- and middle-income countries. In South Africa, cost studies highlighted the importance of HIV prevalence and existing resources as an important determinant in resource use in relation to PMTCT [13, 14]. Overall studies have demonstrated the cost-effectiveness of the transition to and implementation of the Option B+ guidelines. They concluded that Option B+ interventions are inexpensive and compared favourably to other interventions with respect to efficacy [15–19]. Bratt et al. in 2011 assessed annual costs of antenatal care (ANC) including PMTCT, but this was before the Option B+ era [20]. To the researchers' knowledge, as implementation and scale up of Option B+ continues in Uganda, no studies have been conducted to assess costs and cost drivers associated with the provision of Option B+ to mother-baby pairs in Uganda. Therefore, this study set out to determine costs and cost drivers of providing Option B+ services to mother-baby pairs over a two-year period (2014-2015) in selected health facilities in Jinja district, Uganda.

## 2. Methods

### 2.1. Study Setting

**Figure 1:**
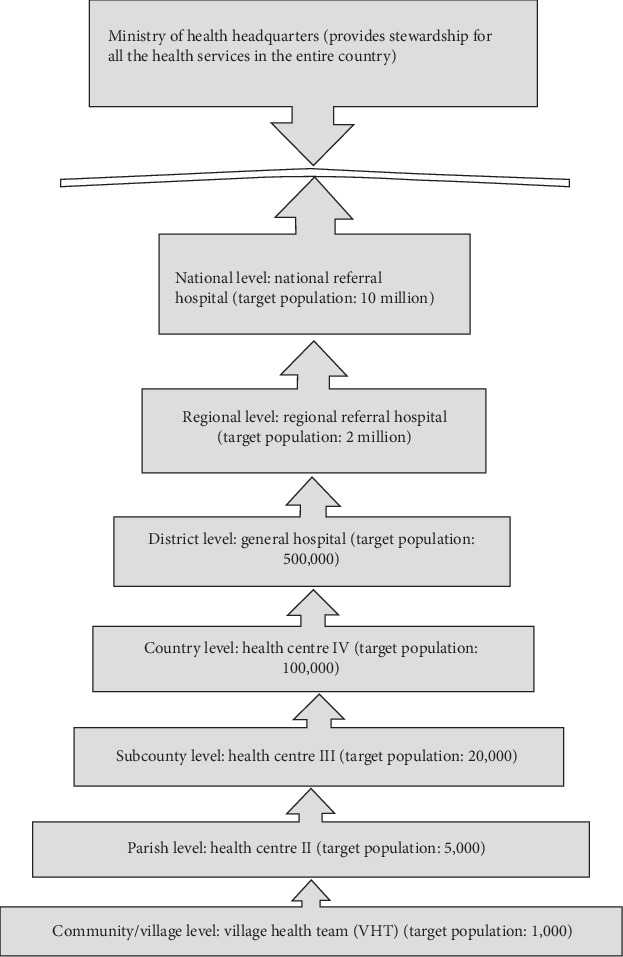
Structure of the health care system in Uganda.

The study was conducted in Jinja district, East-central Uganda, which has a high prevalence of HIV among ANC attendees [21]. The study was conducted in four HC IVs: Bugembe, Mpumudde, Budondo, and Walukuba. These sites were chosen as they had the required data, were among the first facilities to implement Option B+ in the district, and were using the innovative approach detailed below. The HCs were also chosen due to their geographic diversity—they are located in urban (Walukuba and Mpumudde), periurban (Bugembe), and rural (Budondo) areas of the district. All these sites provide PMTCT services. In Jinja district, implementation of Option B+ services started in April 2013. The district uses an innovative interdisciplinary approach engaging healthcare workers (HCWs), village health team members, mentor mothers, and linkage facilitators in the provision of Option B+ services. Mentor mothers are volunteers living with HIV who are trained to provide psychosocial support and health education and to empower pregnant and breastfeeding mothers to improve the health of their families and themselves. Linkage facilitators are campaigners living with HIV who are trained to create awareness and increase uptake of HIV/AIDS services.  Figure 1 shows the structure of the public healthcare system in Uganda [22].

### 2.2. Data Collection

Cost data for providing Option B+ services to mother-baby pairs in 2014 were collected between November 2015 and May 2016 (data were collected retrospectively). Data collection involved conducting face-to-face structured interviews with purposively selected Option B+ service providers, health facility accountants, district officials, and program managers using a structured questionnaire adapted from STAR EC/John Snow Inc. The initial purpose of the survey was the evaluation of outcomes and the impact of the mentor mother model as well as determining the cost-benefit of scaling up the model to the national level [23]. It was administered in English by one of the authors (EB) and trained research assistants (RAs).

Option B+ service providers who participated in the study were drawn from the selected HC IVs while district officials were selected from the district health offices. Program managers who participated in the study were involved in supervising and supporting the HCs in the provision of Option B+ services. They were from the Ministry of Health (MoH) and the two implementing partners, namely, The AIDS Support Organization (TASO) and the Strengthening Uganda's Systems for Treating AIDS Nationally (SUSTAIN) project, who supported implementation of Option B+ in Jinja district. TASO is a nongovernmental organization that offers a comprehensive package of HIV prevention and AIDS care and support services [24]. The SUSTAIN project supports the MoH to strengthen sustainable and innovative approaches for HIV service delivery (including implementation of Option B+ services) at select public healthcare facilities in Uganda. The Option B+ services include high-quality HIV counselling and testing; enrolling HIV-positive women and their infants in care; family support groups for HIV-positive pregnant and postpartum women and their families; integration of family planning into HIV care; HIV-exposed infant monitoring and early infant diagnosis (EID) services; infant and young child feeding in the context of HIV counselling and support; and retention monitoring of HIV-positive women and their infants [25]. Additionally, secondary data were abstracted from health facility accounting records, district payroll records, MoH salary scales, District Health Information Software 2 (DHIS2), and PMTCT registers by one of the authors (JK) and RAs.

Cost categories, parameters, and data sources are summarized in Table 1.

### 2.3. Cost Analysis

All costs were determined from the provider's perspective. All direct healthcare costs and costs of nongovernmental organizations were included, but those incurred by the clients (such as client travel and time costs) were excluded [26]. This enabled the researchers to establish costs borne by the providers and implementers of Option B+ services to mother-baby pairs in Jinja. All costs were converted to USD using the 2014 midyear exchange rate of 2544.6 Ugandan shillings. A time horizon of 2 years was used to cover an antenatal period of six months and a postnatal period of 18 months, after which the mother-baby pairs would be discharged from the mother-baby care point.

Costing was done using the ingredient-based approach, i.e., costing each component of an activity, including capital and recurrent costs [27, 28]. This approach identifies each resource input required and values its market or economic costs [26, 29]. Main ingredients and costs in providing Option B+ services to mother-baby pairs were identified, and a unit cost was calculated. We obtained the mean facility total cost of Option B+ and mean facility cost per cost category by averaging the respective cost over the four health facilities. Overall and cost category mean unit costs per mother-baby pair were calculated as a ratio of respective mean facility costs and average mother-baby pairs per facility. The study estimated program financial costs, reflecting actual costs derived from market prices. All analyses were done using Microsoft Excel.

#### 2.3.1. Allocation of Shared Costs

Costs were allocated as part of cost data analyses following data collection. Direct costs to the PMTCT program were allocated fully to PMTCT. Shared costs were allocated to the MoH coordination office, TASO above-facility coordination and supervision, SUSTAIN transportation of DBS samples from facilities to the regional laboratory/hub, district and facility personnel, facility quality improvement, and overheads from the primary healthcare fund. Coordination and supervision costs were allocated using the proportion of each facility Option B+ clientele to the total district Option B+ clientele for district-level costs and proportion of facility Option B+ clientele to Option B+ national clientele for national-level costs. Facility HIV quality improvement costs were allocated using facility Option B+ clientele as a proportion of total clientele for the HIV program at the facility. Personnel costs for midwives were allocated using Option B+ clientele as a proportion of total ANC attendance, while other facility personnel costs and overheads were allocated using Option B+ clientele as a proportion of total out-client attendance.

#### 2.3.2. Costing of Assets

The cost of the motorcycle used by SUSTAIN to transport DBS specimens was annuitized using an annuity factor of 6.23.

#### 2.3.3. Future Costs

All data on costs incurred by the PMTCT program was determined using 2014 as base year. Costs incurred in the second year (2015) were determined by inflating the 2014 costs by the ratio of 2015 and 2014 USA GDP implicit price deflator [30], as described by Turner et al. [31].

### 2.4. Ethical Considerations

This study was approved by Makerere University School of Public Health Higher Degrees Research and Ethics Committee (protocol number: 308) and Uganda National Council for Science and Technology. Permission to conduct the study was obtained from district and health facilities. Participants were assured of anonymity and confidentiality. All information obtained was kept confidential and used only for study purposes.

## 3. Results

Table 2 shows health facility client parameters. According to data from DHIS2, a total of 50,049 and 1,617 pregnant and lactating mothers in Uganda and Jinja district, respectively, were initiated on Option B+ in 2014. In the same year, 602 pregnant or lactating mothers were initiated on Option B+ in the four HC IVs where the study was conducted. Of these, majority (39.4%, 237/602) came from Bugembe HC IV and Budondo HC IV contributed the least (10.6%, 64/602). Mpumudde and Walukuba HC IVs had 29.7% (179/602) and 20.3% (122/602) mothers, respectively. Infants of these mothers had a final rapid HIV test done from the four health facilities.

Table 3 shows cost parameters and unit costs at the national level and data source.

HIV DNA PCR had the highest unit cost followed by ART for the mother and CD4 cell count test.

Table 4 shows cost drivers, costs, and percentage contribution of cost drivers to the total cost per health facility. The average cost of Option B+ services per facility over 2 years was USD 66,512.7 (range: 32,168.2–102,831.1). The total average 2-year unit cost of Option B+ services per mother-baby pair was USD 441.9 (range: 422.5–502.6).

ART for mothers was the biggest driver of costs (mean contribution: 62.6%; range: 56.0–65.5%). At all sites, the cost of facility personnel was the next highest cost driver (mean contribution: 8.2%; range: 7.7–11.6%), followed by facility-level monitoring and quality improvement (mean percent contribution: 6.0%; range: 3.2–12.3%).

## 4. Discussion

This study determined costs and cost drivers of providing Option B+ services to mother-baby pairs over a two-year period in four HC IVs in Jinja district. Information on costs and cost drivers of providing Option B+ services is vital for policy makers, managers, funders, and implementers to ensure appropriate state and donor fund allocation with clear knowledge of priority areas. This could contribute to the elimination of MTCT of HIV [32].

The study found that the average cost of providing Option B+ services in the second year was higher compared to the first year. The finding could be attributed to the solitary additional cost incurred in the second year to perform the follow-up HIV DNA PCR. The follow-up HIV DNA PCR is done six weeks after an HIV-exposed infant ceases to breast feed [4], which commonly occurs in the second year of provision of Option B+ services to mother-baby pairs.

In our study, the total mean cost per mother-baby pair was USD 441.9 (range: 422.5–502.6) which is congruent with findings from a study conducted in Ethiopia in urban high HIV prevalence health facilities [33]. The Ethiopian study reported that the cost of providing a PMTCT service per woman-infant pair ranged from USD 319 to USD 1099 (2014 cost prices). Correspondingly, another study conducted in Namibia and Rwanda during the pre-Option B+ era found the cost per mother-infant pair in Namibia in the range of USD 203–1030, (2009 cost prices) which is comparable to the current study [34]. The wide range in the cost per mother-infant pair could be due the differences in the PMTCT packages in the Ethiopian and Namibian studies.

Medications, laboratory tests and health facility personnel were found to be the main cost categories in this study.

### 4.1. Medications

ART for mothers was the biggest cost driver, consistent with findings in Côte d'Ivoire where ART cost for Option B+ contributed 68% of the annual treatment cost [35]. Furthermore, our findings are similar to a study done in Ethiopia [33] and a systematic review conducted by Galarraga [36]. The percent contribution in these studies are comparable to those of the current study although there are slight differences in the cost ranges due to possible variations in ART producers and suppliers. The cost of ART is met by the provider and given free to the mothers and their infants. The cost of ART has been significantly reduced over the past decades, but there is still a need for further reduction to prevent ARV shortages [37] which could interrupt provision of Option B+ services. Interruption in the provision of services discourages mothers, which would subsequently result in mother-baby pairs lost to follow-up [38]. ART remains a cornerstone in PMTCT of HIV, and the government should therefore ensure availability of funds to procure sufficient ARVs.

Cotrimoxazole, which plays a key role in preventing opportunistic infections and malaria [39], had a percent contribution of 5.5% to the total mean cost. It is prescribed to HIV-positive mothers and their infants as a prophylaxis. Cotrimoxazole is cost effective in averting opportunistic infections [40–43]. Consequently, higher costs of treating opportunistic infections are averted. The cost found by this study is affordable though slightly higher compared to other literature. A study conducted in Ethiopia with findings closely comparable to the current results combined the cost of all drugs used in Option B+ [33]. Other literature only considered costs of Cotrimoxazole use by HIV-exposed infants, whereas the current study evaluated costs incurred on both mothers and their infants [16, 44].

### 4.2. Laboratory Tests

Significant costs (12.4%) were incurred on laboratory tests. Infant HIV testing (HIV DNA PCR) was the biggest cost driver among the laboratory tests. The cost incurred on the initial HIV DNA PCR was higher than that of the follow-up PCR. This might be attributed to the fact that more mothers bring their infants for the initial PCR as opposed to the follow-up one [45]. Consequently, more initial HIV DNA PCR tests are done compared to follow-up testing. This could be because the initial HIV molecular test is performed when the exposed infant is six weeks old or the earliest opportunity thereafter and coincides with the second immunization visit for the baby and the mother's postnatal care visit [46]. Furthermore, many mothers want to know the HIV status of their exposed infant as soon as possible. The mothers are therefore motivated to do the initial HIV DNA PCR. Studies have reported that some mothers do not return or do not bring their babies with them for subsequent HIV tests if the initial HIV DNA PCR is negative [47].

Monitoring the mothers' CD4 cell counts was a big cost driver. It is important to note that it is not a requirement for a mother to start on Option B+; however, CD4 cell count was the cornerstone in assessing HIV disease progression, making clinical decisions, and monitoring the response to ART [48]. However, since WHO recommended the use of viral load testing as the preferred monitoring tool for people on ART in 2013 [6], many countries have adopted it [49]. Uganda adopted routine viral load testing in 2014 and gradually scaled it up country wide [50]. Viral load testing is more costly than CD4 cell count, and this is anticipated to increase the cost of monitoring mothers on Option B+. Similar to this study, laboratory testing was the second largest cost component of direct costs for providing key services at the facility level to prevent MTCT of HIV in Ghana [51] and in a systematic review by Galarraga et al. [36]. Accordingly, funds should be allocated to sustain laboratory testing.

### 4.3. Health Facility Personnel

Facility personnel costs accounted for 8.2% of the total mean cost per mother-baby pair. Personnel included those at the frontline of delivering care to pregnant and lactating mothers, for example midwives, village health team members, linkage facilitators, and mentor mothers whose role is to ensure smooth implementation of the PMTCT program leading to uptake of Option B+ services and retention in HIV care [52, 53]. In a study conducted in 212 PMTCT facilities in Kenya, Rwanda, South Africa, and Zambia, health personnel costs were reported to be a major cost driver along the PMTCT service cascade [54]. Health personnel play a key role in the provision of Option B+ services. A shortage of healthcare workers is likely to dent the quality of service provision and consequently have a negative effect on patient's adherence to ART and retention in HIV care [53]. This calls for more and sustained funding to cater for the health workforce amidst an increasing number of patients in the era of test and treat.

### 4.4. Monitoring and Quality Improvement

Facility-level monitoring and quality improvement activities accounted for a mean cost contribution of 6.0%. Quality improvement activities are critical in ensuring the provision of standard Option B+ services along the entire PMTCT cascade. This ensures client satisfaction, which potentially leads to uptake of services and retention in care [55]. Indeed, WHO recommends quality HIV care services to achieve desired health outcomes such as the uptake of HIV services, retention in care, and reduction in MTCT and related morbidity and mortality [56].

### 4.5. Coordination and Supervision

A mean percent contribution of 3.8% was incurred on coordination and supervision of Option B+ services. In the current study, above-site coordination and supervision were undertaken by the MOH and the implementing partner (TASO). Proper coordination in the provision of Option B+ services ensures that implementation of the strategy involves all the stakeholders, and is effective and efficient. The Interagency Task Team (IATT) on the prevention of HIV infection in pregnant women, mothers, and their children recommend a well-functioning national coordination mechanism to ensure a successful PMTC program implementation [57]. A well-functioning coordination mechanism is crucial to guide PMTCT program design, implementation, reporting, and monitoring. Studies have highlighted the role of coordination and supervision to address health system challenges in PMTCT programs [58–60]. Ensuring availability of resources to strengthen coordination and supervision is recommended.

## 5. Strengths and Limitations

The study team used program data which gave the real context of providing Option B+ to mother-baby pairs by program implementers. Jinja district has five HC IVs, but the study team failed to access cost data for one of these HC IVs. Nevertheless, the four HCs that were studied gave a representative evaluation. Therefore, this information may be applicable to other HC IVs in the region. However, findings from this study may not be applicable to health facilities that are at a lower or higher level than HC IVs.

## 6. Conclusions and Recommendations

The mean cost for providing Option B+ services per mother-baby pair per health facility was USD 441.9. The three major cost drivers of providing Option B+ services to mother-baby pairs were ART for the mothers, facility personnel, and facility-level monitoring and quality improvement. We recommend that sufficient funds should always be in place to ensure that ARVs are continually in stock, laboratory tests are performed, health facility personnel are remunerated, and quality improvement activities are conducted. In addition, efforts to lower cost of ART for PMTCT would make delivery of Option B+ affordable and sustainable.

## Figures and Tables

**Table 1:** Cost categories, cost items, and source.

Cost category	Cost item	Source
Personnel	Health facility staff	District Health Officer (DHO), accountants, personnel in charge of health facility, facility accounting records, and district payroll records
Medications	ART: tenofovir disoproxil fumarate (TDF)+lamivudine (3TC)+efavirenz (EFV)	ART registers, DHIS2, and National Medical Stores (NMS)^∗^
	Nevirapine syrup	ART registers, dispensing logs, and NMS
	Cotrimoxazole	Dispensing logs and NMS
Laboratory tests	(a) HIV tests (b) HIV deoxyribonucleic acid polymerase chain reaction (DNA PCR) tests (c) Rapid HIV tests (d) CD4 counts	Laboratory registers, DHIS2, NMS, and Gaston Co.
	Dried blood spot (DBS) sample transportation for HIV DNA PCR testing	Laboratory registers, SUSTAIN records, and NMS
Above-site coordination and supervision	MoH coordination	MoH manager, salary scales
	TASO coordination	TASO manager, salary scales
	Option B+ training	DHO, district PMTCT focal person, MoH manager
	Facility-level monitoring and quality improvement	DHO, district PMTCT focal person, and MoH and TASO managers
	Overhead costs (maintenance, utilities, stationery, and fuel)	Accountants, district accountant, personnel in charge of health facility and facility accounting records

^∗^District Health Information System 2 (DHIS2): a free and open source health management data software platform.

**Table 2:** Health facility client parameters in 2014.

Client parameters	Name of HC IV				Total
	Mpumudde	Walukuba	Budondo	Bugembe	
Number of mothers started on Option B+	179	122	64	237	602
Number of ANC attendees	1967	1391	1480	2570	7408
Number of out-patient attendees	22395	36404	18828	28982	106,609
Number of HIV DNA PCR tests done	52	102	42	159	355

**Table 3:** Cost parameters and unit costs at a national level, and data source.

Cost parameter	Unit cost in 2014 USD	Data source
Monthly ART for the mother (TDF+3TC+EFV)	12.0	NMS^∗^
Nevirapine syrup	1.7	NMS^∗^
CD4 test	8.0	NMS^∗^
HIV DNA PCR test	30.0	NMS^∗^
HIV testing (Determine+STAT-PACK test kits)	2.5	Gaston Co.
Testing using Uni-Gold test kit	2.6	Gaston Co.

^∗^National Medical Stores; parameters at the national level including cost of medications, unit costs of laboratory tests (CD4, DNA PCR, and HIV testing), gestation at start of Option B+, useful life of motorcycle (7 years), and the discount rate (3%) were applied to all facility-level cost estimations.

**Table 4:** Cost drivers, mean cost, and percent contribution to total costs of Option B+ services per health facility.

Cost drivers	Mean year 1 costs^∗^	Mean year 2 costs^∗^	Total 2-year cost	Mean cost per mother-babypair	Mean percent contribution (range) %
Personnel					
Facility personnel	2,711.8	2,774.1	5,485.9	36.5	8.2 (7.7–11.6)
Medications					
ART (TDF+3TC+EFV)	20,589.4	21,062.6	41,652.0	276.8	62.6 (56.0–65.5)
Nevirapine syrup	195.4	199.9	395.3	2.6	0.6 (0.5–0.6)
Cotrimoxazole	1,820.6	1,862.5	3,683.1	24.5	5.5 (49–5.8)
Laboratory testing					
HIV enzyme immunoassay (EIA) tests	402.2	411.5	813.7	5.4	1.2 (1.1–1.3)
CD4 counts	1,204.0	1,231.7	2,435.7	16.2	3.7 (3.3–3.8)
1st HIV DNA PCR	1,710.0	1,749.3	3,459.3	23.0	5.2 (2.2–6.7)
2nd HIV DNA PCR	—	337.5	337.5	2.2	0.5 (NA)
DBS sample transportation for HIV DNA PCR testing^∗∗^	593.8	607.4	1,201.2	8.0	1.8 (1.6–1.9)
Above-site coordination, supervision, and training					
MOH	104.4	106.8	211.2	1.4	0.3 (0.2–0.3)
TASO	1,155.4	1,181.9	2,337.3	15.5	3.5 (3.2–3.9)
Option B+ training	135.3	138.4	273.8	1.8	0.4 (0.2–0.4)
Facility-level monitoring and quality improvement	1,973.6	2,019.0	3,992.5	26.5	6.0 (3.2–12.3)
Overheads	115.7	118.4	234.2	1.6	0.4 (0.2–0.7)
Grand total	32,711.7	33,801.0	66,512.7	441.9	100.0%

All costs are in 2014 USD. ^∗^Cost incurred in the second year (2015) were inflated by the ratio of 2015 and 2014 USA GDP implicit price deflator. ^∗∗^SUSTAIN supported transportation of DBS samples.

## Data Availability

The data used to support the findings of this study are available from the corresponding author upon request.
